# Glioblastoma Organoids: Pre-Clinical Applications and Challenges in the Context of Immunotherapy

**DOI:** 10.3389/fonc.2020.604121

**Published:** 2020-12-08

**Authors:** Eliane Klein, Ann-Christin Hau, Anaïs Oudin, Anna Golebiewska, Simone P. Niclou

**Affiliations:** ^1^ NORLUX Neuro-Oncology Laboratory, Department of Oncology, Luxembourg Institute of Health, Luxembourg, Luxembourg; ^2^ Department of Biomedicine, University of Bergen, Bergen, Norway

**Keywords:** brain tumors, glioblastoma, glioma, immunotherapy, preclinical models, organoids, patient-derived xenografts, tumor microenvironment

## Abstract

Malignant brain tumors remain uniformly fatal, even with the best-to-date treatment. For Glioblastoma (GBM), the most severe form of brain cancer in adults, the median overall survival is roughly over a year. New therapeutic options are urgently needed, yet recent clinical trials in the field have been largely disappointing. This is partially due to inappropriate preclinical model systems, which do not reflect the complexity of patient tumors. Furthermore, clinically relevant patient-derived models recapitulating the immune compartment are lacking, which represents a bottleneck for adequate immunotherapy testing. Emerging 3D organoid cultures offer innovative possibilities for cancer modeling. Here, we review available GBM organoid models amenable to a large variety of pre-clinical applications including functional bioassays such as proliferation and invasion, drug screening, and the generation of patient-derived orthotopic xenografts (PDOX) for validation of biological responses *in vivo*. We emphasize advantages and technical challenges in establishing immunocompetent *ex vivo* models based on co-cultures of GBM organoids and human immune cells. The latter can be isolated either from the tumor or from patient or donor blood as peripheral blood mononuclear cells (PBMCs). We also discuss the challenges to generate GBM PDOXs based on humanized mouse models to validate efficacy of immunotherapies *in vivo*. A detailed characterization of such models at the cellular and molecular level is needed to understand the potential and limitations for various immune activating strategies. Increasing the availability of immunocompetent GBM models will improve research on emerging immune therapeutic approaches against aggressive brain cancer.

## Introduction

Among primary malignant tumors of the central nervous system (CNS) the most common and aggressive form is glioblastoma (GBM) with a median survival of 12–15 months (1). Standard treatment of care remained unchanged since 2005, consisting of maximal surgical resection followed by concomitant radiotherapy and chemotherapy with the alkylating agent temozolomide (TMZ) (2). In the last 15 years, novel experimental approaches have shown limited success to improve patient survival and the development of more efficacious therapies remains challenging (3). Several underlying factors, such as aggressive and highly infiltrative growth, inter-patient and intra-tumoral heterogeneity and multiple resistance mechanisms, contribute to the poor outcome (4). More recently, high phenotypic plasticity of GBM has been recognized as an additional hurdle, in particular for precision medicine strategies (5, 6). Improved therapies are desperately needed and novel approaches need to be investigated in adequate preclinical models followed by innovative clinical trials.

Immunotherapy has emerged in recent years as an important success story in oncology, with unprecedented results in various tumor types, e.g., melanoma and breast cancer (7). Rather than targeting tumor cells directly, immunotherapy aims to activate and modulate the immune system in order to stimulate anti-tumor immunity. Currently, numerous clinical trials assess various immunotherapeutic approaches in GBM patients (8). Unfortunately, phase III clinical trials testing immune-checkpoint inhibitors and vaccines have shown so far discouraging results (9, 10). Importantly, GBM is classified as an immunologically ‘cold’ tumor, with limited lymphocyte infiltration, sequestration within the bone marrow and exhaustion of T lymphocytes (11–13). In parallel, GBM induces a highly immunosuppressive microenvironment and features multidimensional immune escape mechanisms. These include the downregulation of MHC Class I molecules, overexpression of immunosuppressive cytokines, activation and recruitment of immunosuppressive cell types, such as myeloid-derived suppressor cells and regulatory T cells (14–16). Although the exact role of resident microglia and blood-derived monocytes remains elusive, tumor associated microglia/macrophages (TAMs) derived thereof largely present a tumor supportive phenotype, which promotes tumor growth, proliferation, and migration (17). This unique GBM tumor microenvironment (TME) will therefore require tailored immunotherapies targeting the immunosuppressive crosstalk within the brain ecosystem, while at the same time stimulating active immunity (18). Currently, a major limitation for the successful development of immunotherapies in GBM is the lack of appropriate pre-clinical models, which recapitulate an adequate immunocompetent environment, along with the accurate molecular and cellular heterogeneity at the tumor and TME level.

For many years, GBM research relied on conventional *in vitro* cell culture systems based on long-term 2D monolayer cell lines grown in serum-containing medium. However, such cell lines do not reflect the heterogeneity of patient tumors, undergo massive clonal selection and genetic drift, resulting in cells that bear little resemblance with clinical tumors (19–21). Hence, translation of *in vitro* studies into the clinic has been challenging, contributing to the failure of clinical trials (22). The adaptation of patient-derived GBM cultures to 3D spheres grown under serum-free conditions, originally developed for neural stem cells, represented a major step forward. In the literature these cultures are also referred to as GBM neurospheres, brain tumor-initiating cells (BTICs) or glioma stem-like cells (GSCs) (term applied in this review). GSCs were shown to better preserve the genetic background of tumors, to maintain a certain degree of phenotypic heterogeneity and molecular gradients (22–24). When implanted intracranially into immunodeficient rodents, they retain invasive growth patterns *in vivo* (25), a feature lost in conventional cell lines. GSCs do not, however, preserve a complex structural tissue architecture including extracellular matrix (ECM) and TME and can be highly proliferative. Since GSCs are generally maintained as long-term cultures, they also suffer to some extent from clonal selection and genetic drift.

Remodeling of GBM tissue architecture and interactions with TME is possible *in vivo* thanks to patient-derived orthotopic xenografts (PDOXs), where patient tumor cells can grow in the rodent brain (26, 27). These are, however, laborious, time consuming and require the use of immunodeficient strains. Since the TME is of rodent origin, molecular and anatomical inter-species differences need to be taken into account. The recent development of 3D organoid cultures has thus emerged as a promising preclinical tool allowing to model complex tumor architecture *ex vivo* whilst at the same time decreasing the use of animals (28). However preclinical drug testing remains challenging for agents aiming at modulating GBM TME, such as anti-angiogenic compounds or immunotherapeutics. Currently, most immunotherapy approaches against GBM are tested *in vivo* using a single syngeneic immunocompetent mouse model (GL261). This murine model displays a hypermutated genome, develops a ‘hot tumor’-like TME and responses to immunotherapies which are of limited clinical value (29–31). In this context tumor organoids integrating immune components along with PDOXs developed in humanized mice emerge as powerful tools for new preclinical studies (32, 33).

In this review we will discuss different protocols for GBM organoid derivation and maintenance, as well as a wide range of organoid-based applications for GBM research and precision oncology. We further review recent attempts in the development of immunocompetent organoids for evaluating immunotherapies and discuss emerging limitations. Finally, we present opportunities and challenges of immunocompetent xenograft models based on orthotopic implantation of GBM organoids in mice with a functional human immune system for studying immunotherapies *in vivo*.

## Organoid Technology for Cancer Modeling

### Healthy Tissue Organoids

During the past decade, growing tissue as organoids *in vitro* has been spearheaded in developmental biology and the technology has been further developed to encompass mature organ tissue (34). Organoids are defined as self-organized, three dimensional (3D) organotypic structures, recapitulating the original organ-like composition *in vitro*. Pioneering work by the Clevers lab successfully established intestinal organoids derived from murine Lgr5+ stem cells, which formed 3D crypt villus structures similar to the *in vivo* organ (35). Nowadays, by applying defined developmental signaling programs, organoids of different organs can be developed. Organoids can be initiated from single or multiple organ-restricted adult stem cells but also embryonic stem cells (ESC) and induced pluripotent stem cells (iPSCs). The denomination of healthy tissue organoids implies several basic features, including the presence of multiple cell types and a morphological organization similar to the parental tissue. They are widely used to model *in vitro* normal organ and disease development, such as infectious, immunological or hereditary disorders (for detailed reviews see (32, 34). Healthy tissue organoids exposed to potential carcinogenic agents, including viral and bacterial infections, are also an excellent model to study early stages of tumorigenesis (36, 37). On the other hand, CRISPR-Cas9 based genetic engineering opened possibilities to assess precise mutational processes at early stages of tumor development (38).

Human cerebral organoids, also called ‘mini-brains’, were established by Lancaster and Knoblich from pluripotent stem cell-derived embryonic bodies (39). Mini-brains developed in ECM (e.g., matrigel) showed characteristics of human cerebral cortex and recapitulated features of different brain regions. Currently, numerous methods are available for the generation of mini-brains, e.g., from pluripotent stem cells (40), from lineage-restricted neural progenitors (41, 42) or from fetal brain tissue (42). Such organoids can also be established to recapitulate region-specific brain structures such as the midbrain (41, 43). Although the presence of different cell subtypes and the maturation stage of brain organoids are limited, they proved to be instrumental in studies of human development and disease (44). They can also be applied for GBM modeling and GBM co-cultures (reviewed below).

### Tumor Organoids

In analogy to healthy tissue organoids, organoid cultures can mimic tumor tissue structure. Several strategies exist to develop tumor organoids: they are generally established directly from resected patient tumors, or can be generated by genetic engineering of stem cells and/or healthy tissue organoids (28). Noteworthy, organoids derived from patient tumor tissue have been used for many years in cancer research and were initially referred to as ‘organotypic tumor spheroids’ (45). At present, the terminology has been updated and terms such as ‘tumor organoids’ or ‘tumoroids’ are in wide use, in analogy to healthy tissue organoids. Instead the term spheroids is now sometimes applied to 3D serum-free sphere cultures, such as GSCs. Protocols and culture conditions for generating patient-derived tumor organoids vary depending on the tumor type. The initial organotypic cultures were derived in serum-containing medium, while more recent protocols apply defined serum-free media similar to healthy organoids. E.g., colon cancer organoids develop in similar conditions as healthy intestinal organoids; however, depletion of Wnt and R-spondins is needed to select for tumor cells (46). Although certain organoids can be developed from single tumor cells after tissue dissociation, the application of intact tumor fragments or multiple cells is recommended to retain genetic and phenotypic heterogeneity. Tumor organoids have been successfully established for many tumor types, including brain (26), breast (47), kidney (48), and liver (49). Interestingly, the success rate of tumor organoid derivation is generally higher than for cell lines and allows to propagate tumors such as prostate cancer (50), less aggressive pancreatic cancer (51) and lower grade gliomas (27), of which cell lines cannot be easily established. This is likely due to minimal clonal selection and a better recapitulation of niche-dependent signals. Compared to previous more simplified *in vitro* models, tumor organoids display a better resemblance with the original patient tumor and retain to a certain extent an *in vivo*-like structural organization (52, 53). If sufficiently proliferative *ex vivo*, organoids can also be successfully expanded into organoid lines with limited clonal evolution, and cryopreserved allowing for efficient and high throughput biobanking (28, 47, 48, 54, 55). This is particularly valuable if combined with corresponding healthy tissue organoids.

## Glioblastoma Organoids

Generation of GBM organoids can be traced back to the pioneering work of Rolf Bjerkvig and colleagues in the 1980ties, who demonstrated the use of patient-derived GBM tissue obtained from needle biopsies or tumor resections to generate multicellular organoids that could be maintained under specific non-adherent culture conditions (45, 56). Although initially termed ‘organotypic tumor spheroids’, these cultures fulfill the criteria of self-organizing organoids. In contrast to 2D or 3D cell lines, these organoids have been shown to closely maintain the genomic profile of the parental tumor, conserve the cellular and molecular phenotype of the original tumor and recapitulate inter- and intra-tumoral heterogeneity (19, 45, 56). In recent years, several research groups have directed their efforts in generating GBM organoids and progress has been made in developing different technical approaches. Here we provide an overview of different available methods and discuss relevant advantages and limitations (**Table 1**).

**Table 1:** Overview and comparison of GBM culture models.

	Culture model	Description	Advantages	Disadvantages	References
Cell lines	2D	Long-term adherent GBM cells cultivated in serum-containing medium	Rapid expansion, low cost, easy maintenance, available for genetic manipulations	Loss of intratumoral heterogeneity, no TME components, clonal selection, genetic drift, *in vivo* phenotype does not reflect human GBM, Low derivation success rate	(20, 21, 24, 57)
	3D	GSC cultures (also termed BTICs), grown as neuropheres in serum-free, growth factor –supplemented conditions	3D growth, moderate expansion, moderate cost, easy maintenance, invasive phenotype *in vivo*, limited recapitulation of molecular gradients, enhanced stem-like features	Clonal selection, some genetic drift, limited intra-tumoral heterogeneity, no TME components, tedious genetic manipulations	(22–24, 58)
Organoids	Patient-derived GBM organoids	Organoids established as primary cultures from resected tumor tissue	High derivation success rate, retention of genetic features and inter- and intra-tumoral heterogeneity, contain some TME components, feasibility of co-culture with autologous immune cells, clinically-relevant drug responses	Costly and labor intensive, lack of vascularization and limited immune component, requires access to fresh patient material and limited by availability of biological material	(25, 27, 59, 60)
	Genetically modified GBM organoids	Gene-edited hESC-derived cerebral organoids initiating tumorigenesis	Recapitulation of early stages of tumorigenesis, defined genetic background, natural development in human brain-like structures, largely recapitulating TME	Time consuming, costly and laborious protocol to generate cerebral organoids, lack of vascularization and immune components	(61, 62)

GSC, glioma stem-like cell; BTIC, brain tumor initiating cell; TME, tumor microenvironment; hESC, human embryonic stem cell.

### Patient-Based Glioblastoma Organoids

**Figure 1:**
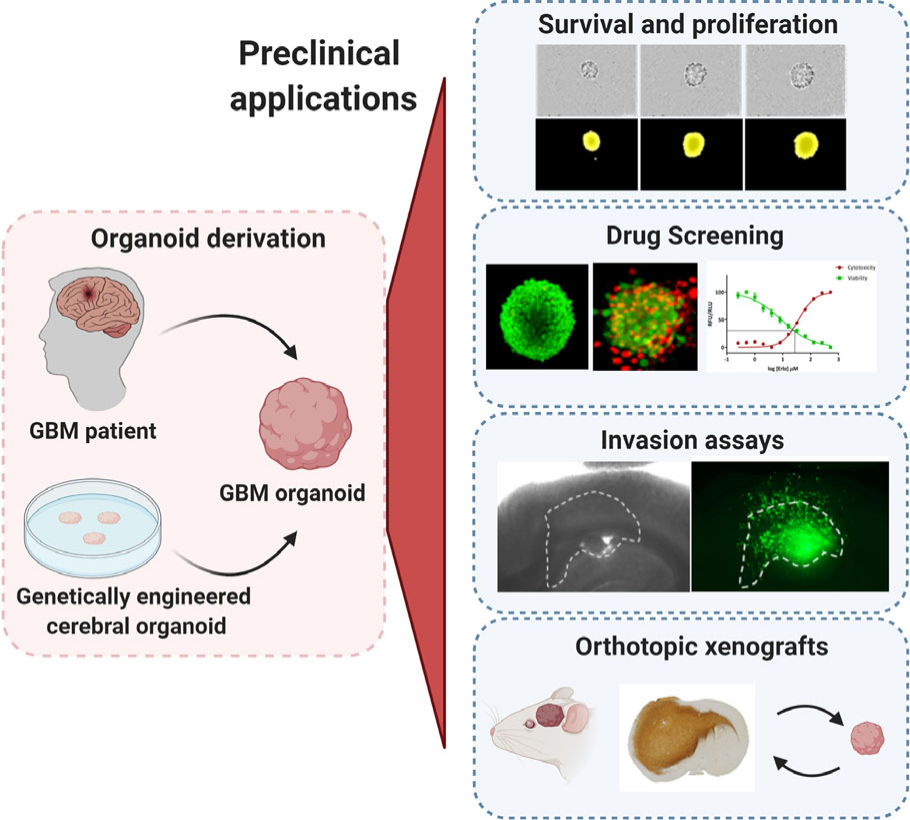
Preclinical applications of GBM organoids. GBM organoids derived from patient tumors or from genetically engineered cerebral organoids can be applied for numerous functional assays such as: tumor cell survival, proliferation and self-renewal, drug screening, *ex vivo* invasion assays, and derivation of orthotopic xenografts. All experimental images were acquired by the authors. Illustration created with Biorender.com.

The Bjerkvig method has been optimized and is still used by multiple labs including ours (25, 27, 63–67) (**Figure 1**). Fresh tumor tissue resected during surgery is mechanically cut in small pieces using scalpels to avoid enzymatic digestion. Tumor fragments are cultured in non-adherent conditions in medium supplemented with serum and non-essential amino acids, but without additional growth factors. During the first days of culture, tissue fragments self-organize into 3D organoids while damaged/necrotic cells are dying. This ensures the preservation of healthy tumor cells within a heterogeneous 3D structure including intact cell-cell interactions and ECM components. GBM organoids generally reach a diameter of 300–1000 µm, thereby recapitulating hypoxic gradients and phenotypic heterogeneity. To a certain extent blood vessels and other TME cells are also retained (25, 66). The success rate of GBM organoid derivation is high (approximately 80%), failure is typically due to excessive necrosis or tissue damage during the surgical procedure (27). To avoid selection processes and genetic drift, we avoid long-term expansion and passaging *in vitro*. Instead we use patient-derived organoids for downstream applications within 1–2 weeks of establishment. The same protocol also allows the derivation of organoids from lower grade gliomas (success rate approximately 70%), recurrent gliomas (27), meningiomas (unpublished) and other brain tumors including metastases (68), but not normal adult brain. Over the last decade we have established a living biobank of over 500 successfully generated patient organoids with an effective cryopreservation protocol.

Our protocol is limited by the availability and the quality of the original patient material*, i.e.*, the resected tumor tissue from surgery or biopsy. Resection of viable tumor tissue followed by fast processing of the sample is essential to maximize viability and to ensure a good organoid quality. GBM organoid growth is limited and variable across patients, where most organoids do not expand beyond the 2-week-culture. They do remain viable for longer time periods and have a tendency to fuse with each other into bigger structures. While for certain patient tumors further expansion and *in vitro* passaging may be possible, we generally do not attempt it in order to limit *in vitro* selection processes. Instead, we apply expansion *in vivo* by implantation of organoids into the brain of immunodeficient rodents. These so called patient-derived orthotopic xenografts (PDOXs, **Figure 1**) enable the propagation of human tumor tissue in a brain microenvironment without relying on *in vitro* expansion. Generally, high quality GBM organoids will efficiently generate tumors in the brain of mice. Serial transplantation, which implies cycles of *in vivo* growth and derivation of organoids from xenotransplanted tumors, allows for extended expansion of patient tumors for large studies, where higher amounts of biological material and/or of xenografted mice are required (27). Similar to patient-derived organoids, organoids derived from PDOXs retain well GBM tissue architecture, ECM and to a certain extent mouse-derived TME (25). We have shown that this procedure preserves genetic and epigenetic profiles of parental tumors, including mutational profiles, copy number aberrations, gene amplifications and DNA ploidy, which are regularly altered in long-term cultures (69). The main challenges to this approach are the costs, the time that is necessary to grow the tumors in the rodent brain and the logistics in planning experiments based on PDOX material. Our model can also be adapted towards growth in serum-free conditions (27, 66) and allows for further derivation of GSC lines. In contrast however to our organoids, we have detected marked changes at different molecular levels during first GSC passages (27).

In recent years, alternative approaches to establish GBM organoids have been published based on serum-free conditions. By combining the protocol of GSC cultures with that used for cerebral organoids, the Jeremy Rich lab successfully established GBM organoids by embedding either dissociated GSCs or intact GSC spheres in ECM (Matrigel) (59). The media composition corresponds to GSC lines and is based on Neurobasal medium supplemented with B27 and the growth factors bFGF and EGF. In contrast to GSC spheres that reach a maximum size of 300 µm after 2 weeks, these organoids can grow up to 3–4 mm in 2 months. Although beyond this point proliferation is limited, organoids remained intact and viable for over one year without passaging. These GBM organoids recapitulated hypoxic gradients, proliferation rates and phenotypic heterogeneity, but do not contain TME components. The organoids give rise to tumors after xenotransplantation, with a longer latency compared to GSCs (59). The protocol can also be applied for fresh mechanically minced tumor tissue or tumors developed in engineered mouse models. No information is available regarding success rate, recapitulation of genetic/epigenetic features of parental tumors and retention of TME when fresh patient material is used.

Another protocol for GBM organoid derivation based on mechanically dissociated GBM tissue was recently reported (60). Small GBM fragments of 0.5–1 mm were cultured in serum-free medium containing mixed Neurobasal/DMEM:F12 supplemented with B27 and N2 and human insulin. Here, growth factors (EGF, bFGF) were not added to the medium. GBM tissue fragments were grown in non-adherent conditions on an orbital shaker without ECM. Under these conditions organoids self-organize within 1–2 weeks and continuously proliferate for over 1 month. To avoid necrosis in the core and to propagate organoids *in vitro*, larger organoids can be regularly cut into small pieces of approximately 0.2–0.5 mm. This allows to preserve cell-cell interactions and natural ECM. These organoids were shown to recapitulate well genetic and molecular traits of original tumors, including inter-patient and intra-tumoral heterogeneity. Phenotypic heterogeneity and a hypoxic gradient were regularly present. Interestingly, despite culture conditions selecting for neural cell lineages, TME components, such as vasculature, TAMs and T cells, were partially preserved within these organoids, at least at early stages. The authors report high success rates of organoid generation from primary GBM (>90%). Recurrent tumors and IDH-mutated astrocytomas also gave rise to organoids, albeit at a slightly lower success rate (75 and 67%, respectively). To create a biobank, primary organoids of approximately 1 month were cut into 100 µm and cryopreserved. Recovered organoids display good viability and continuous growth. These organoids also give rise to tumors upon xenotransplantation with high success rate.

### Glioblastoma Organoid Derivation *via* Genetic Engineering of Cerebral Organoids

GBM organoids can also be generated through genetic engineering of healthy tissue stem cells or cerebral organoids to induce tumor formation. Bian et al. developed an efficient system to introduce simultaneously gain and/or loss of function of tumorigenic genes *via* Sleeping Beauty transposon-mediated gene insertion and CRISPR-Cas9–based mutagenesis of tumor suppressor genes respectively (61). The authors modified the protocol of human ESC-based cerebral organoids (39), where a combination of plasmids is introduced *via* electroporation at the neural stem/progenitor cell stage, before full organoid maturation is accomplished in an ECM. By applying combinations of clinically relevant genetic aberrations they identified sets of genetic cooperations leading to the development of tumor organoids, termed neoplastic cerebral organoids or neoCORs, resembling GBMs and pediatric CNS-PNET. CNS-PNET-like tumors were linked to the overexpression of the oncogene *MYC*, whereas GBM-like cells developed from 3 different sets of genotypes: *CDKN2A* ^–/–^/*CDKN2B* ^–/–^/*EGFR* ^OE^/*EGFRvIII* ^OE^, *NF1* ^–/–^/*PTEN* ^–/–^/*TP53* ^–/–^, and *EGFRvIII* ^OE^/*CDKN2A* ^–/–^/*PTEN* ^–/–^. Emerging GBM-like cells, traced by GFP expression, are proliferative and display classical astrocytic markers. Tumor regions within organoids are visible within one month and show a disorganized structure with marked invasion of GBM-like cells into adjacent normal organoid structures. On the other hand, perivascular palisading necrosis is not present, probably due to the small size and/or the overall lack of vasculature in brain organoids.

Human ESC-derived cerebral organoids were also applied by Ogawa et al. to induce GBM tumors by CRISPR-Cas9–based expression of oncogenic *HRAS* (*HRAS^G12V^*) with simultaneous disruption of the tumor suppressor *TP53* (62). The authors used mature 4-months-old brain organoids and introduced plasmids *via* electroporation to the cortical structures, close to the surface. At 2 weeks after electroporation, first tdTomato-positive transformed cells were visible. At 8 weeks, GBM-like cells encompassed <5% of the organoid; however, onset of fast proliferation leads to complete take over by GBM-like cells by 16 weeks, with the tumor mass growing beyond the boundaries of the organoids. The developed GBM-like cells can be further cultured as adherent GBM cell lines, GSCs, and also form tumors upon xenotransplantation.

### Challenges and Opportunities

Although organoids have gained significant attention in recent years, the technology is still immature. The term ‘organoid’ is broad and encompasses different biological entities based on different underlying procedures. While patient-derived GBM organoid protocols largely converge, they exhibit significant differences, which carry their own advantages and pitfalls. Organoids derived from mechanically minced tissues preserve best cell-to-cell interactions and TME components, whereas organoids derived from dissociated GBM cells may give higher flexibility and reproducibility. Although serum-containing medium is often criticized for inducing differentiation processes, we have not observed this in our short term organoid cultures. Moreover, while serum is known to cause differentiation in normal stem cells, this differentiation process is incomplete and fully reversible in cancer stem cells (5). GBM organoids exposed to serum retain *in vivo* tumorigenicity and heterogeneous expression of stem cell markers similar to patient tumors (5, 27, 66). Moreover limited *in vitro* expansion reduces clonal selection processes and maintains increased tumor heterogeneity. Serum-containing medium however limits proliferation *in vitro* and requires amplification of the tumor material *in vivo*. On the other hand serum-free conditions supplemented with growth factors allow for faster *in vitro* growth, enabling biobanking without the use of animal components, but risk increased tumor cell selection and adaptation of cultures. In general, serum-containing medium better maintains TME components, including glial and immune cells. However such cells were also detected in organoids grown in serum-free medium adjusted for neural cell cultures. More data is needed to fully comprehend the influence of medium components and passaging on the maintenance of tumor and TME populations in organoid cultures. Importantly, both serum-grown and serum-free patient-derived GBM organoids were reported to recapitulate well molecular gradients and phenotypic heterogeneity, which represents a major drawback of GBM cell lines (**Table 1**). In addition, patient-derived GBM organoids largely maintain genetic signatures of their parental tumors, including gene amplifications often lost in GBM cell lines. Still, it remains to be seen to what extent long-term culture of organoid lines may lead to adaptation of tumor cells including clonal selection and further genetic drift, as well as loss of patient-specific genetic and phenotypic heterogeneity. To ensure the accuracy and genetic stability of organoids, the genetic status of organoid lines should be regularly verified after a defined number of passages.

Genetically engineered organoids provide excellent and flexible *in vitro* models for the study of early stages of GBM: they allow for the identification of driver mutations and downstream pathways during the onset of GBM. At present however, it remains unclear to what extent the introduced driver mutations recapitulate the complex genetic heterogeneity of human GBM within the organoid. This has limitations in particular for personalized treatment approaches. Cerebral organoids, particularly those derived from pluripotent stem cells, do not reach complete post-mitotic maturity, and thus represent rather a developing fetal structure than an adult brain. Therefore, they may be more valuable to interrogate tumorigenic potential of pediatric tumors, rather than GBM in adults. Another drawback of this approach is the long process for the establishment of cerebral organoids, which takes months and needs a certain expertise along with a high costs.

## Applications of GBM Organoids

The major asset of organoids is the close recapitulation of genetic and phenotypic heterogeneity of the parental tumor. Hence they hold a great potential for a wide range of pre-clinical applications. In comparison to GBM cell lines, the common drawback of organoids is the increased technical effort needed to perform functional assays and drug testing, particularly in a high-throughput manner. In this chapter, we describe fundamental applications established in the field (**Figure 1**) and review the technical requirements that need adaptation for successful application of organoids to preclinical assays.

### GBM Survival, Proliferation, and Self-Renewal

Assessing GBM proliferation and survival in organoids is more challenging than with conventional GBM cell lines and GSCs due to a compact growth of GBM cells within complex 3D structures. Direct counting of single GBM cells present within patient-derived GBM organoids or after enzymatic dissociation is usually not precise, thus measurement of growth is more often followed by changes in the diameter of the organoids themselves over time (**Figure 1**). To obtain reproducible results, this growth (proliferation) assay should ideally be performed on smaller organoids of similar size at the early development stage to avoid halted proliferation in organoids at later stages. Two options are possible (i): mechanical cutting followed by manual collection of smaller organoids of similar size (60), or (ii) reformation of organoids from dissociated single cells (59). Although our organoid protocol relies on mechanical dissociation of tumor tissue, we showed that the organoid preparation can be adapted for one-off experiments if size standardization is required (25, 27): Organoids can be recreated from enzymatically dissociated patient or PDOX-derived tumor tissue, where single cells self-assemble back into organoid structures. This protocol allows for purification of subpopulations and/or standardization of organoid size and shape for specific functional studies (25, 27, 69–72). This dissociation step should be avoided for serial transplantations and long-term maintenance and propagation of the patient derived tumor material. Self-renewal can be followed by growth of organoids from single cells or *via* serial dilution assay, but these protocols are applicable mostly to the proliferative organoid models based on GSCs (59). Organoid formation and growth can be monitored during a limited period of time in a live cell analysis system or simply by microscopy. Immunohistochemistry-based antibody stainings, classically applied to tumor tissue sections, are a valuable source of information with regard to organoid structure and phenotypic organization. E.g., Ki67 staining can be used to identify proliferating tumor cells. EdU (5-ethynyl-2-deoxyuridine) or BrdU (bromo-deoxyuridine) based DNA labeling assays can be used for the qualitative and quantitative evaluation of proliferation inside organoids (73, 74). Additionally, the estimation of cell death within the organoid can be performed by fluorescent labeling of the cells with viability and cytotoxicity markers allowing for calculation of the ratio between viable and dead cells (70, 72). Proliferation of tumor cells within genetically engineered organoids is possible by detection and quantification of the fluorescence/bioluminescence signal of the genetically modified GBM-like cells (61, 62). Viral barcoding labeling can further enable tracing of clonal lineages and proliferation capacities (75).

### Drug Screening

2D monolayer cell cultures have been widely used for drug screening purposes, mostly because of easy availability and low maintenance costs (76), but unfortunately at the expense of minimal success rates in clinical trials due to lack of efficacy or toxicity. They have been reported to show a disproportionate cellular response to anticancer drugs, partially due to very high proliferation rates and profound phenotypical changes. GSC cultures in combination with novel biological and synthetic scaffolding techniques have shown a better reflection of the patient tumors along with improved drug response when compared to 2D models (22, 77). As these cultures select for proliferative stem-like GBM cells, the drug responses may still be restricted towards these specific phenotypic states. Thus drug responses in heterogeneous organoids may better reflect clinical reality. As all patient-derived GBM organoid models better recapitulate oxygen and nutrient-based heterogeneity, the response to heterogeneous states may be measured simultaneously (27, 59, 60).

Drug responses in GBM organoids can be followed by applying similar technical adaptations as described for proliferation assays, where organoid size and phenotypic/histological changes are measured to determine drug responses. Accurate drug testing requires standardization in terms of size and shape as well as proliferation status. In theory, varying expansion capacities during different stages of organoid development could be exploited to probe drug responses at different proliferation stages of the tumor. Additional challenges need to be taken into account, while adapting drug testing towards high-throughput screens. Classical microscopy-based evaluation is laborious and time-consuming and measurement of organoid size can be limited due to cellular debris surrounding the organoid. Immunohistochemistry for viability, proliferation (Ki67) and apoptotic markers can give a detailed readout on heterogeneous responses to chemo- and radiotherapy within organoid structures (59, 60), but again is low-throughput. The old-fashioned cell viability tests, such as MTT or WST, are not adapted towards non-adherent organoids because of low cell number. Development of more sensitive assays, such as CellTiter-Glo, combined with growth or organoids in 384-well plate format allows to scale up organoid-based drug screen protocols (78) (**Figure 1**). Using GBM PDOX-derived organoids of standardized size derived from 1000 tumor cells, we have applied a similar medium-throughput protocol and showed patient-specific and clinically relevant responses to TMZ and EGFR inhibitors (27). In accordance with clinical outcome, *MGMT* promoter-methylated GBM organoids showed higher sensitivity to TMZ in comparison to *MGMT* promoter-unmethylated organoids, an effect that is not always recapitulated in GBM cell lines (79). Similarly to short term GBM cultures (80), patient-derived GBM organoids’ responses to EGFR inhibitors were linked to EGFR expression and mutations present in individual tumors. These associations cannot be easily assessed in long-term adherent cell lines and GSCs due to the general loss of *EGFR* amplification in these cultures (81). Clinically-relevant heterogeneous responses were also observed in patient-derived organoids cultured in serum-free medium (60), although more models in (epi)genetically-defined groups will be needed for a comprehensive evaluation. We were also able to reconstruct GBM organoids in alginate using cell printing technology. Cell printing combined with automated high content imaging of viable cells allows for a higher throughput automated drug library screening (77, 82). Other detection techniques, such as optical metabolic imaging, not requiring specific dyes for detection also arise as an interesting option (83).

Although the organoid technology is very promising and enables relatively fast drug testing in clinically relevant timing, several challenges should be considered. Similarly to nutrients and growth factors, drugs may not be able to fully penetrate bigger 3D structures, thus organoids of smaller sizes should be applied for drug testing. Both patient-derived organoids and genetically-engineered organoids contain also normal non-malignant cells to various degrees, thus more adequate read-out techniques may be needed to distinguish effects on different cell types. *E.g.*, Brian et al. quantified the ratio between tumor and normal cells *via* flow cytometry (61). Although fluorescence/bioluminescence allows to distinguish GBM-like cells within genetically-modified organoids, high throughput application may require faster detection and precise calculation algorithms. An additional drawback in GBM is the lack of equivalent patient-derived normal brain organoids. This would allow to screen for drugs that selectively kill tumor cells while leaving healthy cells untouched. Although iPSCs could be derived from each GBM patient and used for cerebral organoid development, the technology is still immature and inefficient to be applied for routine testing. This could be partially resolved by applying GBM organoids in co-cultures with cerebral organoids or organotypic brain slices, as described for the invasion assays below (74).

### GBM Invasion

Tumor cell invasion is a hallmark of GBM strongly contributing to inevitable regrowth of tumors after surgery (84). Invasion capacities of tumor cells are classically tested *in vitro* with Boyden chambers, where single cells can invade membrane pores covered with different combinations of ECM. Subjecting intact organoids to Boyden chamber assays is not optimal as invasion from a 3D structure through a membrane is irregular and difficult to measure. Although single cells obtained from enzymatically dissociated organoids can be applied (25), this may lead to an additional stress of GBM cells not adjusted to survive as single cells. The sprouting assay represents a more adapted approach as it simply involves embedding organoids directly in the ECM and quantifying cells invading out of the organoid into the matrix (65, 67). A more advanced technique for measuring invasion could take advantage of adult organotypic brain slice cultures of rodent or human origin (**Figure 1**), where organoids encounter the natural brain microenvironment (85, 86). So far this technique was applied to GBM cell lines and GSCs, injection of organoids into the brain slice may be more challenging. Organoids may remain non-attached or only adhere to the surface of the brain slice. Importantly, this technique requires fluorescent labelling of tumor cells for detection and quantification of invasion and single cell velocity by microscopy.

Organotypic brain slice cultures can also be replaced by healthy cerebral organoids. In this case, direct co-culture is possible, where GBM cells can spontaneously fuse with brain organoids to form hybrid organoids. Linkous et al. showed successful interactions between GSCs with human ESC and iPSC-derived brain organoids, creating a so called GLICO (cerebral organoid glioma) model (74). The authors showed that GSCs were able to invade and proliferate within the healthy brain organoid and to form interconnecting microtubes. Another study confirmed that GSCs transcriptionally adapted to mini-brain microenvironment in line with their *in vivo* behavior (87). Although Linkous et al. showed similar invasion of GSCs in cerebral organoids of different age, others reported GBM cell invasion only in early stage cerebral organoids, whereas invasion into fully mature organoids was halted (88). Similar co-cultures of human cerebral organoids could be applied to patient-derived GBM organoids in the future. Of note, the protocol is tedious, as efficient GBM invasion inside the cerebral organoid often requires removal of the ECM embedding (87, 88). If ECM is preserved, GBM cells primarily adhere to the matrix and grow on top of the surface. Injection of GBM organoids inside the cerebral organoids could also be envisaged, although this may destroy the fragile mini-brain structure, particularly if still embedded in the ECM. A similar co-culture approach is also feasible with mini-brains derived from neural progenitors isolated from rat fetal brain (42, 65). Patient-derived GBM organoids and GSCs spontaneously fuse with rat brain organoids and progressively invade the healthy brain tissue. The process is faster and more efficient in comparison to human cerebral organoids, as rat brain organoids are not embedded in ECM. Also other brain tumors were shown to interact with healthy rat brain organoids, but GBM cells showed most prominent invasion, up to complete destruction of the healthy tissue (89). Although species differences should be considered, rat brain organoids derived from fetal tissue are faster to generate, more reproducible and appear to reach better maturation status compared to human cerebral organoids derived from iPSCs or ESCs. Again, fluorescent or bioluminescent labelling is needed to efficiently measure GBM invasion into brain organoid structures. Similarly, invasion can also be followed directly in genetically-engineered organoids as GBM-like cells develop naturally within the intact cerebral organoids (61, 62). Although cerebral organoids allow for easier access to human brain structures, they miss critical structures required for invasion. In particular, it is well known that GBM cells preferentially infiltrate along vascular structures (90), which are not present in cerebral organoids. The inclusion of vascular elements into cerebral organoids (91, 92) in co-cultures with GBM organoids, as well as directly into genetically engineered GBM organoids, could therefore be a valuable tool for future studies on the dynamics of GBM invasion along blood vessels and developing invasion inhibiting treatment strategies.

### Patient-Derived Orthotropic Xenografts

Patient-derived xenografts (PDXs) represent a well-established preclinical cancer model allowing for propagation and investigation of human tumors in immunodeficient rodents. Classically PDXs are derived by subcutaneous implantation of patient tumor tissue fragments, with a take rate of around 50% for GBM tumors (93). In case of specific organs, such as brain, patient-derived orthotopic xenografts (PDOXs) better recapitulate tumor histopathological features and TME. Implantation of tissue fragments directly into the brain is technically challenging and may lead to unreproducible tumor growth. Thus application of patient-derived GBM organoids for implantation ensures technical feasibility and standardization, while avoiding GBM selection and adaptation. In general, the majority of GBM organoids of different culture models give rise to tumors upon xenotransplantation in the brain and recapitulate well histopathological features of patient GBMs such as invasion and angiogenesis. To obtain consistent tumor take and growth rates, we implant six to ten intact GBM patient-derived organoids into the brain of immunodeficient mice or rats respectively (25, 27, 94, 95). We have shown that organoids derived from high grade gliomas, including IDH mutant astrocytomas and GBM, are able to grow in the brain with very high rate of tumor take (27).. Successful engraftment and PDOX propagation for > 3 *in vivo* passages was obtained for 86% of GBMs (35/41) and 25% grade III gliomas (2/8). Failure of GBM organoid engraftment was attributed to initial poor organoid quality, whereas no association between organoid quality and tumor take was seen for grade III gliomas. The *in vivo* tumor latency strongly depends on the parental tumor and can vary from several weeks to several months. Organoids from treatment naïve and treated GBMs can develop and give rise to tumors *in vivo*. We have been also able to generate paired longitudinal models from tumor samples collected at different timepoints from the same patient, thus recapitulating disease progression over time (27). Such models are invaluable tools to study tumor evolution and treatment resistance in a personalized *in vivo* setting. In case of more proliferative GBM organoids cultured in serum-free conditions, implantation of a lower number of intact organoids (down to 1 organoid/implantation) was sufficient to develop tumors *in vivo* (60). The authors reported successful engraftment of 8 organoid cultures derived from 7 patients and tumors were visible 1–3 months after implantation. Hubert et al., applied enzymatic dissociation of organoids prior to implantation (59). Although no exact tumor take rates were reported, implantation of GSC-derived organoids should be highly efficient. Interestingly, despite containing a similar number of self-renewing cells GBM organoids showed longer latency than implanted GSCs of the same patient (59). Genetically-engineered GBM organoids were also shown to give rise to intra-cranial tumors *in vivo* (no tumor take reported, mean survival 90–100 days after implantation of 3*10^5^ cells) (62) or expand in renal capsules (17/20 neoCORs, 85%) (61).

PDOXs allow for the propagation of tumor material *in vivo* (live biobanking) within an adequate brain microenvironment including structural (vasculature, blood brain barrier), cellular (neurons, glia, microglia/macrophages) and metabolic components (cerebrospinal fluid, brain interstitial fluid). This procedure allows also to avoid long-term culture and expansion of organoids *in vitro*. GBM organoids can be further obtained from established PDOXs and serially transplanted to maintain the patient tumors over multiple generations (27). We showed that organoid-derived PDOXs remain stable across generations in mice, recapitulate histopathological features of human GBM, with various level of angiogenesis, necrosis and invasiveness (25). Such PDOXs represent invaluable patient ‘avatars’ for downstream experimental needs and applications (**Figure 1**). Applications range from *in vivo* drug validation studies, protocol optimization for magnetic resonance imaging (MRI), the use of isotopic tracers for dynamic profiling of tumor metabolism *in vivo*, genetic and phenotypic analysis, to identification of novel biomarkers and therapeutic targets (5, 95–100). We showed that anti-angiogenic treatment in organoid-derived GBM PDOXs leads to clinically relevant responses with no survival benefit (70, 95). This is in contrast to observed GBM cell line-derived xenografts, which show strong dependence on angiogenesis to survive *in vivo* (101). Monitoring of PDOX by MRI allows to follow ‘patient avatars’ in a similar fashion as in the clinical setting and to complement drug testing on organoids (27, 70). This includes the visualization of an intact or disrupted blood brain barrier (BBB) in the various tumor compartments, an essential component of the GBM TME. Because of the selective permeability of the BBB regarding blood derived molecules (102), PDOXs are essential to validate the therapeutic effect of novel treatment strategies in a meaningful preclinical *in vivo* setting to avoid failure in the clinical phases.

Because human tumors need to be engrafted in immunodeficient rodents, limitations of PDOXs include the lack of a complete immune system and potential interspecies incompatibilities at the molecular level. Importantly, our previous studies showed that human tumor cells can functionally interact with cells from the TME in PDOX despite interspecies differences, e.g., rodent endothelial cells form aberrant blood vessels (25) and are affected by anti-human VEGF treatment (70, 95). Similarly, myeloid cells are present in PDOXs and are modulated by the tumor graft (27). Since they represent the major immune cell type of the brain TME, targeting the immunosuppressive nature of myeloid cells can be tested in PDOX (17, 103). Nevertheless, while the innate immune system is largely intact in nude mice, the lack of lymphocytes prevents certain applications for modern immunotherapy. This can be overcome on the one hand by the generation of immunocompetent GBM organoids for *ex vivo* studies and on the other hand, by the establishment of PDOX models in humanized mice for *in vivo* studies.

## Immunocompetent Organoid Culture—Which Immune Cells to Use?

The interactions between immune and tumor cells critically influence the onset, progression and treatment of human malignancies. Although the brain has been for long considered as an immune privileged organ, it is clear that the immune system plays a key role in development and surveillance of brain homeostasis (104). Nevertheless, the brain remains an immunologically distinct site, which is also reflected in the TME of brain tumors (105). TME includes brain resident and infiltrating myeloid cells, natural killer cells, dendritic cells and regulatory T cells, classifying GBM as strongly lymphocyte depleted tumors (13). A major challenge of all current GBM organoid models remains the establishment of an intact TME including the immune cell compartment.

**Figure 2:**
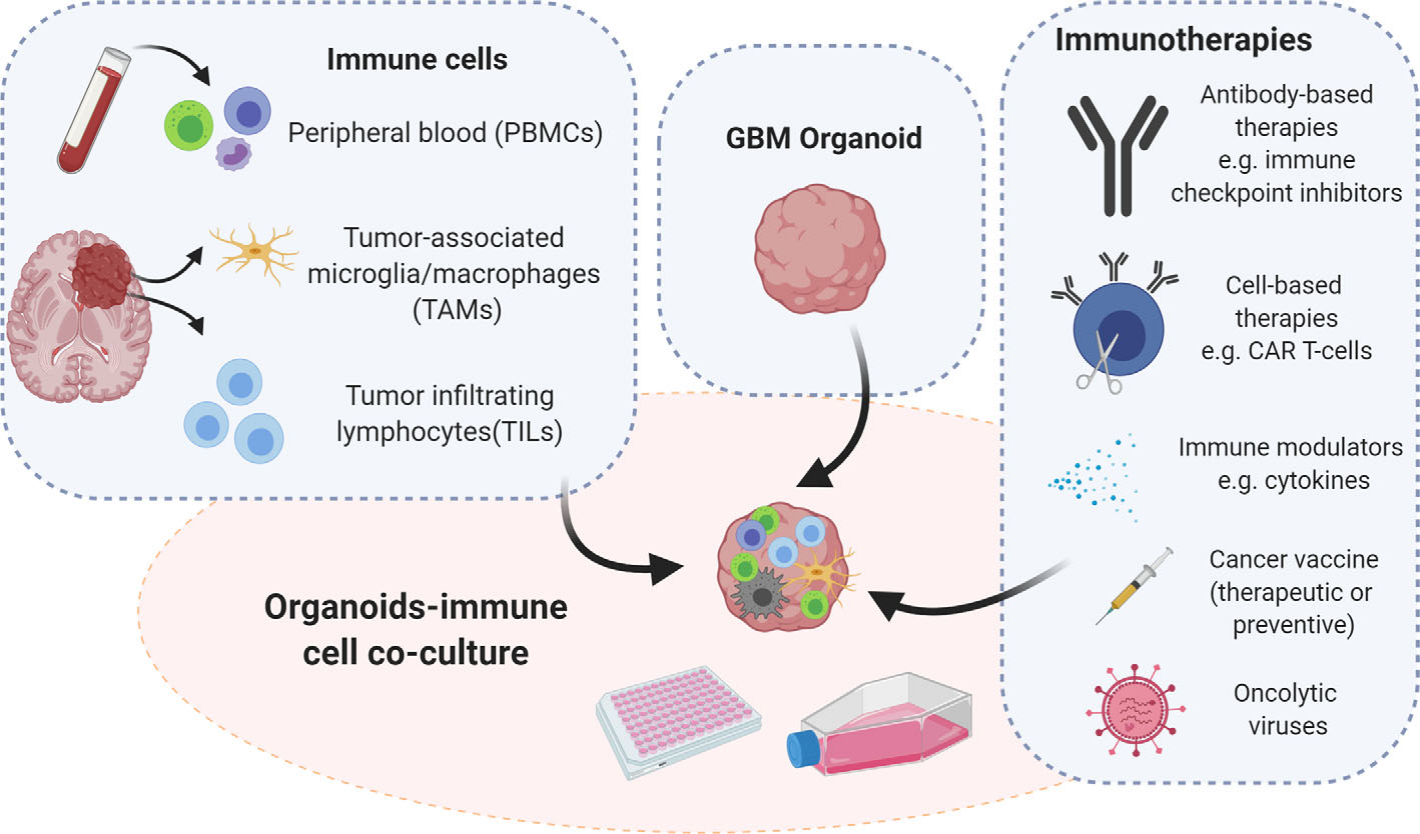
Strategies for immunocompetent GBM organoid development. Immunocompetent organoids can be set up as co-cultures of tumor organoids with immune cells derived either from the tumor itself or from peripheral blood of patients or healthy donors. Immunocompetent organoids are applicable to functional assays and therapeutic intervention studies, which implicate assessment of tumor-immune cell interactions. Illustration created with Biorender.com.

The establishment of immunocompetent cancer organoids is an active field of research and an urgent need. Such novel models fill a gap in pre-clinical research, allowing for functional and translational studies for immunotherapies and promoting the investigation of tumor-immune cell interactions (106). Considering the high demand for personalized immunotherapy, immunocompetent *ex vivo* models present a promising platform for individual patients, by advancing the development of new immunotherapeutic strategies. Here we provide an overview of protocols employing various immune cell populations for the setup of immunocompetent tumor organoids that could be applied to GBM modelling. Tumor organoids can be co-cultured with different immune cell populations depending on the origin of the immune compartment. Immune cells can be isolated from the periphery or directly from the tumor site (**Figure 2**). Opportunities and limitations of both are discussed below.

### Peripheral Mononuclear Cells

To mimic the immune microenvironment, immunocompetent organoids can incorporate autologous or allogeneic immune components in the culture. In the case of autologous immune components, cells are isolated from the same patient who provided the tumor tissue to generate tumor organoids. Allogeneic immune cells imply a non-self-source, such as healthy blood donors. The main source of relevant immune cells are peripheral mononuclear cells **(**PBMCs), which comprise lymphocytes (T cells, B cells, and NK cells) and monocytes. Isolated PBMCs should not contain neither granulocytes (neutrophils, basophils, and eosinophils) with multi-lobulated nuclei, nor nuclei-free erythrocytes and platelets. PBMCs can be cultured as a bulk population or individual immune cell populations can be further isolated through magnetic separation or FACS, cultivated and expanded as monocultures.

#### Autologous Peripheral Mononuclear Cells

Patient blood presents a valuable source to obtain patient’s own immune cells in the form of autologous PBMCs. PBMCs are easily accessible and can be obtained through a simple blood withdrawal prior to surgery when the tumor tissue is removed. This allows to establish matched immunocompetent organoids for individual patients. Promising results with *ex vivo* co-cultures of organoids with autologous PBMCs have been reported for non-small cell lung cancer and colorectal cancer (107, 108). A proportion of co-cultures with organoids positive for MHC class I led to the activation of T cells, which were able to eliminate tumor organoids, but left non-neoplastic organoids from the same patient unaffected. No responses were observed for MHC class I deficient tumors. In this protocol organoids were dissociated into single cells and adapted to lymphocyte medium prior to the co-culture (107, 108). This proof of concept study suggests that tumor reactive T cells can be expanded from peripheral blood and activated by matched tumor organoids. Activated T cell populations can thus be used subsequently to test cytotoxic properties *ex vivo* and/or to analyze the T cell receptor repertoire. Ultimately, effector T cells displaying immune reactivity after co-culture with tumor organoids could also be applied for adoptive cell transfer, if a sufficient number of T cells is generated.

Such systems have not yet been reported for GBM and it remains to be seen whether GBM cells will trigger an immune response and immunogenic properties in autologous PBMC-based organoids, particularly in case of MHC I deficiency. Multiple studies have shown that GBM patients’ blood presents peripheral T cell lymphopenia (low T cell counts) and a high number of myeloid-derived suppressor cells (12, 15, 109). This is further exacerbated by corticosteroids (dexamethasone), a treatment often provided upfront to reduce tumor-associated edema and improve clinical symptoms. Therefore the timing of blood withdrawal is crucial and should ideally be conducted before surgery and before any other treatment is given. Additional technical issues need to be taken into account (i): pre-stimulation of tumor organoids with interferon γ (IFNγ) may be needed to enhance antigen presentation (ii), pre-stimulation of T cells with anti-CD28 and interleukin-2 (IL-2) may be required to support proliferation and expression of anti-Programmed cell death 1 (anti-PD1) thereby counteracting Programmed cell death ligand 1 (PDL1) inhibitory effects on tumor cells (107).

#### Allogeneic Peripheral Mononuclear Cells

Allogeneic PBMCs are isolated from the blood of healthy donors. In contrast to autologous PBMCs, they represent normal blood with appropriate cell counts and were never exposed to tumor-associated stimuli released into the peripheral system. Allogenic PBMCs have been extensively used to obtain purified immune cell populations (T cells, NK cells, monocytes), which were applied to co-cultures with conventional tumor cell lines. Although activation of immune cells in 2D cultures appears rather straightforward, patient-derived 3D systems add additional challenges linked to immunosuppressive factors such as hypoxia and high lactate levels (110) as well as potential HLA incompatibilities. Co-cultures of allogenic blood components have not yet been reported for GBM organoids. Tang et al., developed sophisticated co-cultures of macrophages with GSCs using a bioprinting method (111). Macrophage cultures were obtained from a monocytic cell line (THP-1), human iPSCs or PBMCs from healthy donors. Co-cultures were embedded in hyaluronic acid rich hydrogels, representing a main component of GBM ECM. Additional cellular components such as astrocytes and neural stem cells, could be incorporated to the embedded co-cultures. Of note, unpolarized M0 macrophages successfully interacted with GSCs and polarized towards a protumoral M2-like macrophage phenotype.

### Tumor Derived Immune Cell Populations

#### Tumor Infiltrating Lymphocytes

Tumor infiltrating lymphocytes (TILs) present within resected tumor fragments represent another source of lymphocytes. Isolation of TILs can be performed simultaneously during tumor tissue processing, which facilitates biobanking regulations and protocols, e.g., no additional blood withdrawal from the patient is necessary. In contrast to systems using peripheral immune cells, TILs allow for the *ex vivo* modeling of the TME-intrinsic immune responses. Cells present within the TME are enriched for populations already instructed by the tumor, hence they may not need further activation to produce the desired tumor-intrinsic phenotype. Co-cultures with TILs are particularly important for interrogating immune checkpoint expression on tumor cells and TILs and determining tumor-specific efficacy of checkpoint inhibitors. The main disadvantage lies in low number of TILs obtained from most tumors. Compared to metastatic melanoma where TILs are frequently isolated in high numbers and applied in adoptive T cell transfer, enrichment of tumor-reactive T cells in gastrointestinal cancers was more challenging (112). These limitations can be partially overcome by using organoids derived from mechanically processed tumor tissue, where TILs are naturally preserved. E.g., Neal et al. showed that patient-derived tumor organoids from different type of cancers, including melanoma, renal and non-small cell lung cancer preserve endogenous TILs and other TME components (113). Here tumor organoids were embedded in a collagen matrix and subjected to an air-liquid interface set-up. TILs and other TME components were present for up to 2 months within tumor organoids. TILs remained functional and triggered a cytotoxic response upon PD1/PDL1 checkpoint blockades. Although TIL survival was prolonged by IL-2 or anti-CD3/anti-CD28, further optimization will be needed for long-term preservation. Another study reported the maintenance of CD45+ immune cells for up to 8 days within epithelial tumor organoids (114). A protocol applying co-cultures of tumor organoids and separately isolated TILs was reported for rectal cancer, where TILs were able to interact with tumor organoids embedded in the ECM and to partially restore cytotoxic activity upon (anti-PD1) treatment (115).

Establishing a co-culture system for GBM organoids and TILs will be technically challenging due to the low number of infiltrating lymphocytes in GBM. TME components were reported to be present within patient-derived GBM organoids derived from tissue fragments including a small fraction of T cells and TAMs (60, 66). However, similar to epithelial cancer organoids, the TME compartment is progressively lost over time and separate TILs may be needed for long-term experiments. The situation in GBM is further complicated by the fact that a large fraction of infiltrative T lymphocytes represent regulatory T cells rather than tumor-directed cytotoxic T lymphocytes, promoting an immunosuppressive TME (116). Thus co-cultures and manipulation of TILs towards a different phenotype will be of particular importance for GBM-specific immunotherapies. Finally, since isolation of TILs from tumor tissue requires enzymatic dissociation, it interferes with the derivation of GBM organoids from mechanically cut tissue fragments. In this case GBM tissue will have to be sub-divided for TIL isolation and GMB organoid derivation, compromising the number of T cells and organoids obtained per patient.

#### Tumor-Associated Microglia/Macrophages

TAMs play an important role in GBM biology and are known to facilitate tumor growth and invasion. TAMs originate from both microglia and blood-derived monocytes, and acquire a strong immunosuppressive phenotype in GBM (17, 117). GBM display a prominent infiltration of TAMs which represent the majority population of non-neoplastic cells (40–50% of the non-tumor cell mass), thus they can be isolated from tumor tissue resected during surgery. This is generally based on selection of CD11b positive cells with FACS or MACS followed by subsequent cultivation. This is a laborious method which generally results in low yields, which is complicated by the fact that TAMs do not generally proliferate in culture. Culture and freezing conditions should be optimized in order to keep the viability at a high level. Similar to TILs, TAMs are also partially preserved in GBM organoids, allowing for direct investigation of tumor-TME crosstalk during early stages of organoid culture.

### Modified Immune Cells (CAR-T Cells, CAR-NK Cells)

CAR-T cells are genetically modified T cells expressing a chimeric antigen receptor (CAR) on their surface, which results in the binding to specific antigens on tumor cells leading to tumor cell killing. As patient-derived tumor organoids retain well specific antigens and heterogeneity, they appear as an advantageous model for *ex vivo* testing of CAR-T cell therapies. Jacob et al. demonstrated the utility of patient-derived GBM organoids to test adoptive T cell therapy *ex vivo* (60). EGFRvIII is a constitutively activated EGF receptor mutant that is overexpressed in a large number of GBM. CAR-T cells engineered to react with EGFRvIII expressing cells were co-cultured with GBM organoids with differential EGFRvIII expression levels. CAR-T cells were able to invade GBM organoids and expansion of EGFRvIII-specific T cells was observed within organoids with high EGFRvIII levels. Specific CAR-T cell mediated toxicity was further observed towards EGFRvIII positive cells, as evidenced by an increased cleaved-caspase 3 signal and increased presence of granulated T cells in close proximity of EGFRvIII positive apoptotic cells. This proof-of-concept study demonstrated the capacity of patient-derived organoids as an *ex vivo* test bed for immunotherapy. Unfortunately the clinical situation remains more complex and a recent pilot trial with EGFR-targeting CAR-T cells did not achieve a meaningful clinical effect (118).

In addition to T cells, NK cells can also be engineered to express CARs. In a study with patient-derived colorectal cancer organoids, CAR-mediated cytotoxicity was investigated using a CAR-NK cell line (CAR-NK-92 cells), which represents a less laborious source for CAR-engineered immune cells. CAR-NK-92 mediated cytotoxicity against tumor organoids was observed at low levels of tumor associated antigen expression, whereas it was absent against healthy colon organoids (119).

### Important Considerations and Optimization Steps

An increasing number of reports present protocols for derivation and maintenance of immunocompetent tumor organoids, demonstrating their utility to model the immune microenvironment and study the effects of immunotherapies (32). Although initial promising studies of immunocompetent GBM organoids were reported, further development and optimization of protocols is needed. The experimental settings for the establishment of immunocompetent GBM organoids may depend on several factors, including the research question at hand, the availability of autologous blood and the amount of available tumor tissue. Limited or unviable tumor tissue obtained from surgery is a common problem, which limits the amount of tumor organoids and TME cells that can be isolated. This is particularly challenging if tumor organoid and TIL isolation requires dedicated tissue pieces and preparation protocols. Another challenge is the timing of the co-culture set up with cells from the same patient. While establishing GBM organoids takes 1–2 weeks, blood or tumor derived immune cells are ready on the day of collection. Since the expansion of these cells is either limited (TAMs) or should be avoided (lymphocytes) and/or the cells cannot be easily maintained in culture, a proper cryopreservation and thawing process is critical for the use of viable immune cells at later time points. Furthermore, as indicated above, the recovery of T cells from GBM patients either from the tumor tissue or from PBMCs is expected to be low because of limited T cell infiltration and peripheral T cell lymphopenia, respectively, characteristic of GBM patients (12, 109).

Another challenge is to establish optimal culture conditions for all co-cultured cell types. This includes medium composition, duration of the co-culture, the immune-tumor cell ratio, and the read-out for cytotoxic responses. Co-cultures are generally performed in the immune cell-specific medium which may compromise GBM organoid viability and may not reflect brain physiology. Culture conditions need to be adapted to different GBM organoids and immune cells under investigation. The ratio between tumor and immune cells depends on the effector cells applied in the study. Generally, a target to effector ratio of 1:10 to 1:20 is reported for PBMCs (108). With specific subset of immune cells, such as CAR cells, less effector cells are required (60, 119). Whether or not the organoid is dissociated prior to co-culture also impacts tumor-immune cell interactions. Spontaneous infiltration of immune cells into intact tumor organoids may be particularly challenging if ECM is applied for organoid derivation (107). In addition, the use of a rodent-derived matrix may lead to unspecific activation of immune cells against foreign antigens. Finally, it remains to be seen whether GBM cells display sufficient immunogenicity, which requires large numbers of neo-antigens and appropriate antigen presentation capacity to induce an active immune responses. Antigen presenting cells, such as dendritic cells or TAMs are potentially needed to enhance the tumor-T cell interactions in the culture.

## Immunocompetent *In Vivo* Tumor Models in Humanized Mice

PDOXs derived in immunodeficient rodents are gold standard preclinical models for drug efficacy *in vivo* studies in oncology (120). Yet, the lack of a fully functional immune system limits their use for testing immunotherapies. Hence, the generation of PDOXs in humanized mice appears as a promising immunocompetent *in vivo* system recapitulating patient-derived tumors and immune compartment (33). Since the first description of humanized mice in 1988, a plethora of protocols has been developed (121, 122). Generation of humanized mice requires a highly immunodeficient mouse background to obtain efficient engraftment of a hematopoietic human system. Thus, the NOD.Cg-Prkdc^scid^ Il2^rgtm1Wjl^/SzJ (NSG) strain is frequently applied. NSG mice lack mature T, B cells and hemolytic complement, the Il2^rgtm1Wjl^ mutation prevents cell signaling through multiple cytokines leading to a lack of NK cell activity. Moreover, the polymorphism of the *signal regulatory protein α* (*sirpα*) allele in the NOD background allows a functional ‘do-not-eat-me-signal’ between mouse myeloid cells and human CD47, while the deficiency in Prkdc^scid^ confers sensitivity to radiation (123, 124). Currently two main approaches are in use to reconstitute the human immune system (i): HU-PBMC model applying PBMCs isolated from human adult blood or (ii) HU-CD34 model based on human CD34+ hematopoietic stem cells (HSCs).

### HU-PBMC Model

The HU-PBMC model can be derived by intravenous, intraperitoneal or intrasplenic injection of human PBMCs from adult donors into adult NSG mice (>8 weeks old). This model allows a fast and efficient engraftment rate with approximately 15% of human CD45+ cells constituting blood in mice after one week and up to 50% of human CD45+ cells 4 weeks after inoculation. The human CD45+ fraction is mainly composed of mature human T cells with a higher level of CD4+ rather than CD8+ cells (125). Thus, this model is specific to T lymphocytes and is not suited for investigating monocytes, which remain mostly mouse-derived. The main advantages are the fast engraftment of human cells and the possibility to implant PBMCs and tumor cells from the same patient, avoiding HLA mismatch. Unfortunately, the model can only be applied short-term, as PBMCs undergo human thymic education and present human MHC leading to an immune reaction against mouse MHC, known as Graft versus Host Disease (GvHD), and death of the mice after approximately 4 weeks (125). Because of the short experimental window (3 weeks) the HU-PBMC model is generally difficult to adapt to *in vivo* tumor development protocols. NSG mice with a double knock out for MHC Class I and II (NOD.Cg-Prkdc^scid^ H2-Ab1^em1Mvw^ H2-K1^tm1Bpe^ H2-D1^tm1Bpe^ Il2rg^tm1Wjl^/SzJ) can be applied to extend the experimental window. In the absence of mouse MHC, this transgenic strain allows up to 100 days for tumor development monitoring (126, 127). Ashizawa et al., took advantage of NSG MHC I/II KO mice to develop subcutaneous GBM xenografts in a HU-PBMC model with PBMCs obtained from the HLA-partially matched donor (126). One day after X-ray irradiation of mice and PBMC injection, the U87 GBM cell line was implanted subcutaneously, which allowed tumor development in the experimental time frame of the humanized model. The authors report a successful response to anti-PD1 treatment. It should be noted that MHC knock out may impact mouse microglia functionality, which was not investigated in this study. As described in this study, preconditioning irradiation can be applied in these mice to increase the percentage of human cell engraftment. This is not recommended in the NSG strain, where it will lead to a faster development of GvHD.

**Figure 3:**
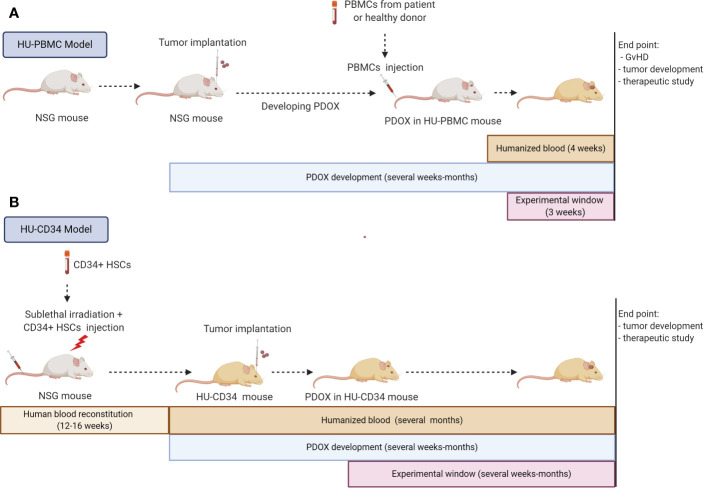
Strategies for immunocompetent GBM PDOXs development in humanized mice. Immunocompetent GBM PDOXs can be generated in HU-PBMC or HU-CD34 mice. The experimental schedule and therapeutic window depends on the humanized model applied and tumor development time **(A)**. Due to the short survival of HU-PBMC mice, tumor implantation should precede the PBMC injection. The best time point will depend on tumor latency. The experimental window is limited due to development of Graft versus Host Disease (GvHD) within 4 weeks, depending on the genetic background of the NSG mice **(B)**. HU-CD34 model requires more time for the generation of humanized blood which is counterbalanced by the longer survival of the mice (>1 year). The tumor implantation timepoint and experimental window depend on the tumor latency and need to be synchronized according to the required readout. Illustration created with Biorender.com.

So far no GBM PDOX model was reported in HU-PBMC mice and it is currently not clear to what extent HU-PBMCs will translocate to the mouse brain. PDOX development in the mouse brain can take from several weeks to several months, often going far beyond the 4–10 weeks before the GvHD. To overcome this issue, PBMCs could be injected after the tumor is well established which would also avoid potential tumor cell rejection due to the brain surgery-induced inflammation (**Figure 3**). In this case, X-ray irradiation should be avoided not only because of increased GvHD, but also because of its impact on tumor growth. The implantation protocol requires 10x10^6^ human PBMCs per mouse, which may be challenging to obtain from GBM patients, which display severe lymphopenia. *Ex vivo* expansion of T cells/PBMCs from patients or healthy donors with partial HLA match could overcome the T cell limitation. The application of HLA-partially matched PBMCs from healthy donors would also allow to expand humanized studies to previously established GBM PDOXs, for which patient blood is not available.

### HU-CD34 Model

HU-CD34 mice are created from human CD34+ hematopoietic stem cells (HSC) isolated from umbilical cord blood, bone marrow, fetal liver or mobilized PBMCs. HSCs are injected intravenously, intrafemoraly, or intrahepatically into freshly irradiated new born or young NSG mice (<3–4 weeks old). After 12–16 weeks, up to 25% of CD45+ cells in mouse blood represent human cells and mice can be used for experiments (**Figure 3**). This provides a much wider experimental window for implantation of tumor cells. The reconstitution of the human hematopoietic system (human/mouse ratio and maturation level) differs depending on the mouse strain and the organ (128–130). HU-CD34 NSG mice reconstitute well B and T cells but a low level of myeloid lineage cells is seen in the blood. These mice are able to survive for more than a year, with a relatively stable ratio of human/mouse cells in the blood. The partial incompatibility of growth factor signaling required for hematopoiesis explains some developmental or functional defects observed in myeloid cell differentiation or maturation of T cells (128, 131, 132). Additional injection of human growth factors (133) or application of transgenic strains expressing several human growth factors can improve maturation of human immune cells (125). For example, the NSG-SGM3 (NOD.Cg-PrkdcscidIl2rgtm1WjlTg(CMV-IL3,CSF2,KITLG)1Eav/MloySzJ) triple transgenic mice expressing human Stem cell factor (SCF), Interleukin-3 (IL-3) and Granulocyte/macrophage stimulating factor (GM-CSF), enhance the number of T cells and myeloid cells (134). It is currently unclear if sublethal irradiation, necessary prior to CD34+ HSC implantation, can affect microglia functionality in the mouse brain. Interestingly, it has been reported that HU-CD34 NSG mice can present human HSC-derived microglia/macrophage-like cells integrated with mouse microglia in the brain (135). A specific transgenic strain producing human IL-34 (NOG-hIL34 mice) further improved development of human microglia/macrophage-like cells in the brain (136).

The HU-CD34 model has been successfully combined with several cell line-derived xenografts and PDXs of different cancer types (137–139). Although a perfect HLA match between CD34+ HSC donor and tumor patient is impossible, a partial HLA match did not negatively affect tumor growth in recent reports on PDXs (139, 140). HLA loss is a well described escape mechanism in many tumors, including GBM, which may compensate the possible HLA mismatch (141, 142). Moreover, as human immune cells mature through the mouse thymus according to the mouse MHC I and II, human T cells are not fully functional and do not reject human tumor cells with different HLA (143). An alternative BLT (Bone marrow, Liver, Thymus) model, which applies the co-transplantation of fetal liver and thymus from autologous CD34+ HSCs donors, allows for improved development of HSCs and their positive selection through human MHC. The functionality of T cells is improved, yet in this situation the partial HLA match leads to higher incidence of GvHD (144, 145).

For GBM PDOXs the HU-CD34 model appears as a preferred model than HU-PBMC, because of the improved reconstitution of human immune cells and the longer experimental window. GBM PDOX developed in HU-CD34 mice would recreate most comprehensively a functional human immune system, allowing for *in vivo* therapeutic interventions targeting tumor-immune cell crosstalk. So far only one study described GBM orthotopic xenografts developed in HU-CD34 model. Zhai et al., have successfully implanted U87 cells and GBM cells derived from two subcutaneous PDX models into the brain of HU-CD34 BTL (146). The presence of human T and myeloid cells was confirmed in the blood, lymph nodes, spleen, as well as within the tumors developed in the brain, but not in normal adjacent brain. It remains to be seen if a similar reconstitution of the human hematopoietic system is present in GBM PDOXs that develop over longer time periods and if these humanized PDOXs recapitulate clinical features of GBM patients, such as lymphopenia, leading to decreased amount of human T cells in the blood.

## Conclusion and Perspectives

In recent years not much progress has been made to improve survival of GBM patients and treatment options are still very limited. The technology of tumor-treating fields is the only recent treatment modality, which provided a positive outcome in a phase III clinical trial, but has its own inherent drawbacks that are debated in the community (147). Since TMZ, no novel drug has been developed that led to prolonged patient survival (148). This failure can at least be partially attributed to inappropriate pre-clinical models, which do not fully recapitulate GBM, hence novel physiologically relevant models are urgently needed. Organoid culture models have emerged to complete the scientific toolbox. Patient-derived GBM organoids and GBM organoids derived from genetically engineered human brain organoids have been successfully established and have been shown to better recapitulate GBM genetic and phenotypic characteristics in comparison to 2D GBM cell lines and 3D GSCs. Although technologically more challenging, GBM organoids represent a promising and exciting pre-clinical model and are a powerful tool to foster our understanding of GBM biology and an emerging platform for drug screening. If established from a patient-derived system, these organoids offer an approach for personalized medicine, prompting to better predict treatment responses for patients. Due to the relatively quick generation time of patient-derived organoids, *ex vivo* studies are being conducted in a reasonable and clinically relevant time frame and could ultimately guide clinical decisions. Technical challenges need to be addressed in future studies and further improvements to incorporate an adequate TME are warranted. Immunocompetent GBM organoids, based on co-culture with either tumor or blood-derived immune cells, will be crucial to bring forward novel immunotherapeutic approaches. We anticipate that future studies will incorporate immunocompetent organoid cultures in their experimental design to investigate not only immune-tumor interactions, but also to investigate current and novel immunotherapies, such as adoptive T cell transfer, immune checkpoint inhibitors or oncolytic viruses. Moreover, PDOX generated in humanized mice will provide another important tool essential to improve drug development and preclinical testing *in vivo*. Such developments and improvements of pre-clinical models should have a major impact on preclinical research and clinical studies and eventually on patient care.

## Author Contributions

EK, AG, and SN contributed to conception and design of the manuscript. EK wrote the first draft of the manuscript. AC and AO wrote sections of the manuscript. AG and SN corrected different versions and finalized the manuscript. All authors contributed to manuscript revision, read, and approved the submitted version.

## Funding

The authors are grateful for the financial support of Télévie-FNRS (grants GBModImm no. 7.8513.18 and TETHER no. 7.4615.18).

## Conflict of Interest

The authors declare that the research was conducted in the absence of any commercial or financial relationships that could be construed as a potential conflict of interest.
